# Supplementary figures and images for: Pretectal neurons control hunting behaviour
Source: eLife. 2019 Oct 8;8:e48114. doi: 10.7554/eLife.48114 (PMC6783268; doi:10.7554/eLife.48114)

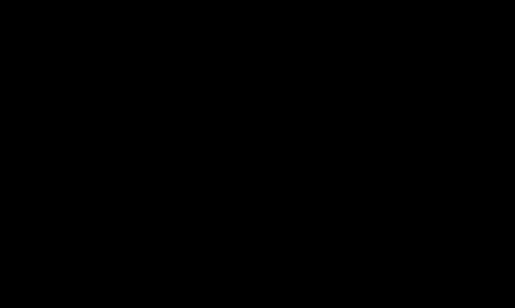

Supplement: Supplementary file 2. — TIFF stack containing binary mask defining the ‘avOT’ anatomical region, in ZBB space. [file elife-48114-supp2.tif]

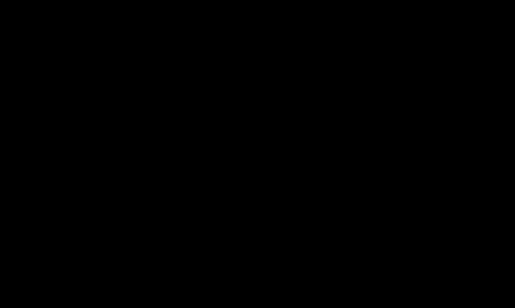

Supplement: Supplementary file 3. — TIFF stack containing binary mask defining the ‘AF7-pretectum’ anatomical region, in ZBB space. [file elife-48114-supp3.tif]

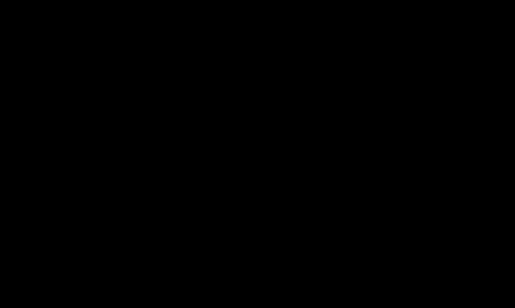

Supplement: Supplementary file 4. — TIFF stack containing binary mask defining the ‘NI chata’ anatomical region, in ZBB space. [file elife-48114-supp4.tif]
